# Supplementary material for: Predicting visual function by interpreting a neuronal wiring diagram
Source: Nature. 2024 Oct 2;634(8032):113–23. doi: 10.1038/s41586-024-07953-5 (PMC11446822; doi:10.1038/s41586-024-07953-5)
Supplement: Supplementary file 6 — CRF and ERF predictions for individual TmY4 and TmY9 cells. Analogous to Supplementary Data 3, but for TmY target types. Shown are the top four monosynaptic pathways, the strongest pathway passing through each of the top ten intermediary types (ranking from Extended Data Fig. 7), and the trisynaptic pathway Tm1–TmY–Dm3–TmY (see the section entitled Prediction of spatial normalization). [file 41586_2024_7953_MOESM6_ESM.zip › DataS4/TmY9qΓèÑ/720575940632783564.pdf]

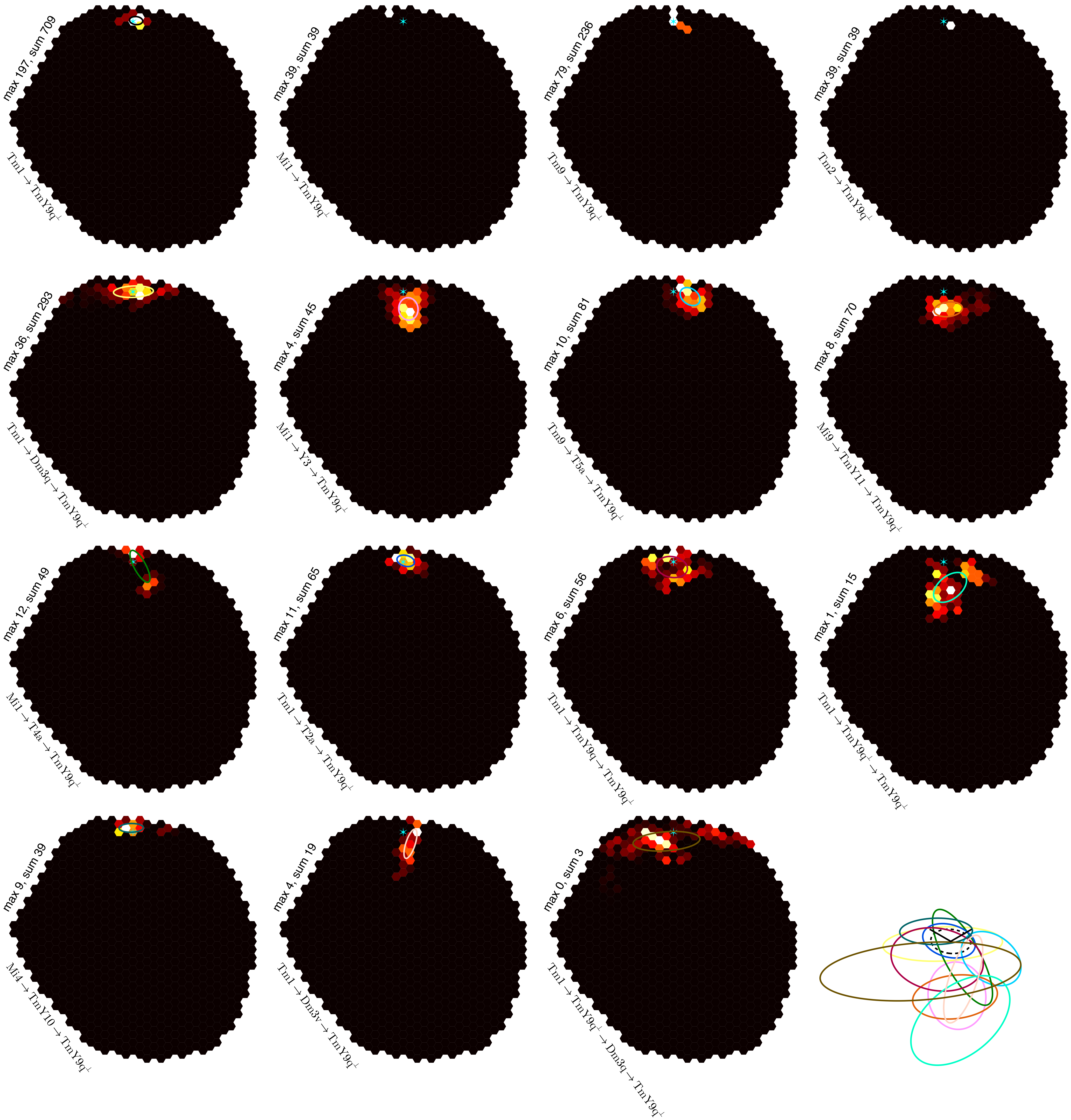
max 197, sum 709
Tm1 → TmY9q⊥
max 39, sum 39
Mi1 → TmY9q⊥
max 79, sum 236
Tm9 → TmY9q⊥
max 39, sum 39
Tm2 → TmY9q⊥
max 36, sum 293
Tm1 → Dm3q → TmY9q⊥
max 4, sum 45
Mi1 → Y3 → TmY9q⊥
max 10, sum 81
Tm9 → T5a → TmY9q⊥
max 8, sum 70
Mi9 → TmY11 → TmY9q⊥
max 12, sum 49
Mi1 → T4a → TmY9q⊥
max 11, sum 65
Tm1 → T2a → TmY9q⊥
max 6, sum 56
Tm1 → TmY9q → TmY9q⊥
max 1, sum 15
Tm1 → TmY9q⊥ → TmY9q⊥
max 9, sum 39
Mi4 → TmY10 → TmY9q⊥
max 4, sum 19
Tm1 → Dm3v → TmY9q⊥
max 0, sum 3
Tm1 → TmY9q⊥ → Dm3q → TmY9q⊥
